# The Human Virome: Viral Metagenomics, Relations with Human Diseases, and Therapeutic Applications

**DOI:** 10.3390/v14020278

**Published:** 2022-01-28

**Authors:** Geng-Hao Bai, Sheng-Chieh Lin, Yi-Hsiang Hsu, Shih-Yen Chen

**Affiliations:** 1 School of Medicine, College of Medicine, Taipei Medical University, Taipei City 11031, Taiwan; b101105010@tmu.edu.tw; 2 Department of Education, Taipei Medical University Hospital, Taipei City 11031, Taiwan; 3 Department of Pediatrics, School of Medicine, College of Medicine, Taipei Medical University, Taipei City 11031, Taiwan; jacklinbox@tmu.edu.tw; 4 Department of Pediatrics, Division of Allergy, Asthma and Immunology, Shuang Ho Hospital, Taipei Medical University, New Taipei City 23561, Taiwan; 5 Beth Israel Deaconess Medical Center and Harvard Medical School, Boston, MA 02215, USA; yihsianghsu@hsl.harvard.edu; 6 Broad Institute of MIT and Harvard, Cambridge, MA 02142, USA; 7 Department of Pediatrics, Division of Pediatric Gastroenterology and Hepatology, Shuang Ho Hospital, Taipei Medical University, New Taipei City 23561, Taiwan

**Keywords:** metagenome, virome, disease, SARS-CoV-2

## Abstract

The human body is colonized by a wide range of microorganisms. The field of viromics has expanded since the first reports on the detection of viruses via metagenomic sequencing in 2002. With the continued development of reference materials and databases, viral metagenomic approaches have been used to explore known components of the virome and discover new viruses from various types of samples. The virome has attracted substantial interest since the outbreak of the coronavirus disease 2019 (COVID-19) pandemic. Increasing numbers of studies and review articles have documented the diverse virome in various sites in the human body, as well as interactions between the human host and the virome with regard to health and disease. However, there have been few studies of direct causal relationships. Viral metagenomic analyses often lack standard references and are potentially subject to bias. Moreover, most virome-related review articles have focused on the gut virome and did not investigate the roles of the virome in other sites of the body in human disease. This review presents an overview of viral metagenomics, with updates regarding the relations between alterations in the human virome and the pathogenesis of human diseases, recent findings related to COVID-19, and therapeutic applications related to the human virome.

## 1. Introduction

A wide range of microorganisms are found in the human body, including viruses, bacteria, archaea, fungi, and protozoa. The communities of microorganisms and their interactions both with one another and the host have strong impacts on human health and disease [1]. The virome is the viral fraction of the microbiome, and it is dominated by bacteriophages that infect bacteria as well as eukaryotic viruses that infect human cells. Major factors shaping the human virome include diet, breast milk, medications such as antibiotics and immunosuppressants, host genetics, cohabitation, geography, underlying diseases, and aging [2]. Nowadays, most microbiome studies have focused on bacteria due to the availability of powerful techniques for investigating bacterial communities. However, the development of high-throughput nucleic acid sequencing technology has facilitated the detection, identification, and characterization of viruses in the human virome. Previously, detection and discovery of viruses required virus propagation in cell culture. Shotgun sequencing was first applied to the analysis of viral populations in the environment in 2002 [3]. Several cost-effective approaches have since been developed, including metagenomic next-generation sequencing (mNGS), to identify known or novel microorganisms via the sequencing of DNA or RNA from samples. Increasing numbers of viral metagenomic studies have used similar methods to identify so-called “dark matter,” i.e., previously uncharacterized viruses [4]. More novel viruses have been discovered and added to existing databases. The National Center for Biotechnology Information Genome database till December 2021 contained 11,545 complete viral genome sequences [5]. Although this still represents only a small fraction of all viral genomes, the continuous updating of public sequence databases is expanding the sensitivity of virus identification platforms.

The human virome consists of viruses colonizing the human body by replicating in human cells and those infecting the prokaryotic and eukaryotic components of the human microbiome [6]. In addition to viruses that can cause infection, virome dysbiosis is thought to be related to several human diseases. In the coronavirus disease 2019 (COVID-19) pandemic, a large proportion of people infected with the causative virus, severe acute respiratory syndrome coronavirus 2 (SARS-CoV-2), were asymptomatic, whereas others developed severe disease. In addition to well-known risk factors, virome dysbiosis was also shown to influence the severity of outcomes of SARS-CoV-2 infection. Studies have uncovered a potentially important role of the human virome in persistent SARS-CoV-2 infection and preventing reinfection [7]. Moreover, virome-related therapeutic applications to overcome dysbiosis, such as fecal microbiota transplantation (FMT) and phage therapy, have been applied in several clinical trials. Potential adverse effects related to each treatment type should be taken into consideration, and clinical protocols are also required for such applications during the COVID-19 pandemic and post-COVID-19 period [8].

Several recent articles have documented studies of the human virome, including reviews of the use of metagenomics for pathogen detection [9,10,11]. Several overviews of viral metagenomic pipelines have also been reported [4,12,13,14]. Moreover, the human gut, the most studied site in the body, has been shown to contain a remarkably varied virome. Several studies have summarized interactions between the gut virome and human diseases [15,16]. However, viromes at other sites within the body also play roles in human diseases. The COVID-19 pandemic will likely change our approach toward human viromic studies. Here, we review the utility of the viral mNGS approach in identifying and characterizing viruses, the composition of the human virome, relationships of human diseases with gut as well as oral and respiratory viromes, and therapeutic approaches related to the human virome.

## 2. Metagenomic Approach

### 2.1. Viral Metagenomic Approach

Several metagenomic approaches have been developed to sequence total nucleic acid contents in a sample. Shotgun sequencing and subtraction prior to sequencing are often used for functional and taxonomic characterization. Virus concentration has been used for novel virus discovery, and hybridization capture for low-copy-number sequences is also commonly employed [9]. Next-generation sequencing (NGS) allows many DNA fragments to be sequenced simultaneously and independently. The application of NGS to clinical microbiological testing has led to the development of mNGS, which has the advantage of unbiased sampling that allows the identification of known microorganisms, unexpected pathogens, and even new organisms. mNGS can also be coupled to targeted approaches, such as those using primers for conserved 16S ribosomal RNA (rRNA) regions and microbiological detection via internal transcribed spacer (ITS) sequences. With mNGS, genomic information for evolutionary tracing, strain identification, and the prediction of drug resistance can also be provided [11]. Metagenomics has clinical applications in a wide variety of areas, such as antimicrobial resistance, the microbiome, human host gene expression, and oncology [17].

**Figure 1:**
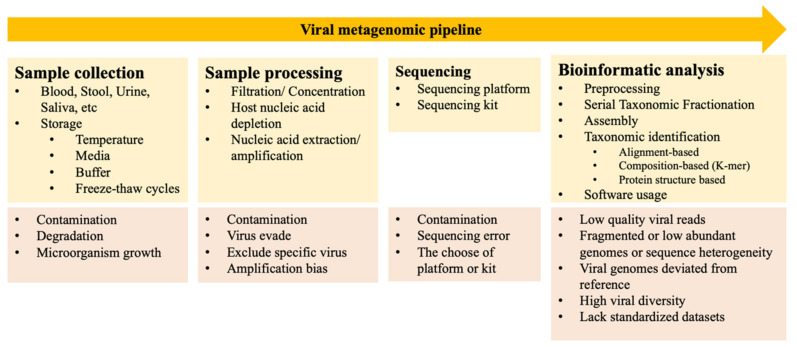
Workflow of viral metagenomic approach. The viral metagenomics pipeline usually includes sample collection, sample processing, sequencing, and bioinformatics analyses. Sample collection methods and the choice of storage temperature, media, and buffer vary across different sample types. Sample processing before sequencing includes sample filtration and/or concentration and nucleic acid amplification. Sequencing technology includes Nanopore (long-read data) and Illumina (short-read data). Bioinformatics analysis includes preprocessing, serial taxonomic fractionation, assembly, and taxonomic identification. The flow chart shows the drawbacks and potential for bias at each step of viral metagenomic analysis [4,12].

Viral metagenomic pipelines include sample collection, sample processing, sequencing, and bioinformatic analyses (Figure 1) [4,12,13,14]. To collect viruses from various samples, viral particles are concentrated, contaminating nucleic acids are eliminated, and viral nucleic acids are extracted, amplified, and purified [18]. Sample collection methods differ according to the type of sample, such as blood, stool, urine, or saliva. The major factor that affects the recovery of viral nucleic acids and viral community information is the sample storage conditions, including storage temperature, types of media and buffers used, and whether the samples undergo freeze–thaw cycling [12]. Each processing step is accompanied by risks of contamination and nucleic acid degradation, which may compromise downstream analyses [4]. Sample processing is thus a crucial step in viral metagenomics. Protocols for the purification of virus-like particles (VLPs) vary in the required amount of starting material, buffers used, and filter pore size. Following filtration, samples are centrifuged to remove any debris and further concentrate VLPs [19]. Host nucleic acids can also be depleted by adding chloroform to disrupt cell membranes when working with samples that have a high level of host contamination. Methods for nucleic acid extraction and the amplification of viral nucleic acids are designed to enrich viral sequences [12]. However, the loss of viruses that evade purification, the loss of some transcripts, and amplification bias in these steps may skew the virome profile [4]. Once samples have been collected and processed, they are subjected to sequencing. When it comes to single-molecule sequencing technologies, Nanopore detects DNA sequences by using a nanopore. It can generate full-length genome haplotypes due to its advantage of long-read sequence. The long nanopore reads have a huge benefit in de novo genome assembly. As for short-read sequence technique, Illumina is a second-generation sequencing that detect DNA by using reversible dye terminators. It can allow the detection of minor variants; however, it does not permit the complete viral haplotypes reconstruction [20]. The numbers of viruses detected are determined by the type of virus, sequencing platform, and sequencing kit(s) used [12]. Viral metagenomic sequencing is different from sequencing cellular organisms as viruses do not contain universally conserved genomic sequences such as 16S rRNA in bacteria. Moreover, sequencing errors may lead to the false identification of viruses or variants, and sequences may be difficult to classify due to the lack of matches in current virus databases [4].

Computational pipelines, i.e., protocols for processing and analyzing NGS data, include this series of steps: preprocessing/quality control, filtering of sequences, assembly, taxonomic identification, validation, and analysis [4]. Since raw reads are often low in quality, preprocessing removes sequence adapters and low-quality and low-complexity regions [11]. Serial taxonomic fractionation (STF) allows the successive separation of reads into taxonomic groups, such as humans, fungi, or bacteria, to improve the speed of downstream steps and the accuracy of viral taxonomic assignments [4]. Assembly involves assembling sequences from one organism with the assumption of even coverage into larger contigs that represent viral genomes. One such method is reference-based assembly, which requires the reference genome as reading alignment, and another is de novo assembly, which relies on determination of the connections between each read and all others. Increasing the length of genome assemblies could increase the chance of obtaining a significant alignment when performing taxonomic identification and increase the efficiency of manual curation and organism analysis [4]. Finally, several methods have been developed for taxonomic identification: alignment-based classification methods such as BLAST, Bowtie, and Diamond rely on nucleotide alignments from updated sequence databases and are highly sensitive but slow and resource intensive; composition-based classification involves annotation based on k-mer matching and nucleotide frequencies and is fast but less sensitive for the identification of divergent sequences [4]; protein structure-based approaches using open reading frames (ORFs) to identify novel viral genomes; and reference- and alignment-independent machine-learning methods, such as DeepVirFinder, that identify viral sequences in metagenomics using deep learning [21].

### 2.2. Biases and Challenges Associated with Viral Metagenomics

Although viral metagenomics has allowed us to understand the complexity and richness of the human virome, including bacteriophage and other virus populations, there are still some challenges and biases not only at each step of viral metagenomics but also in the preparation of viral reference databases. First, it is important to consider contamination from sample collection and processing when interpreting virome data. Second, fragments of viral genomes are often less abundant than those of the host, bacteria, or other microorganisms in clinical metagenomes. Hence, the observed viral genomes often deviate from reference genomes, and high viral diversity may lead to ambiguous sequence reconstruction. Furthermore, regarding software application, there is a lack of standardization in test datasets used across multiple studies [14]. Furthermore, the majority of viruses have yet to be grown in culture, and it is not clear which organisms serve as hosts for replication of many members of the virome. Moreover, virome analysis mainly focuses on DNA sequencing, with only a few RNA phages reported. Given that the bacterial microbiome was the main objective of past metagenomic studies, as RNA is less stable in samples typically used for metagenomics, elucidation of the actual composition of RNA viruses in the virome may be limited for technical reasons. Last but not least, a large number of unclassified viral sequences await characterization. These uncharacterized viruses are called “dark matter”. Although more annotated genomes are added to databases, and the advanced pipelines have been developed to process virome data, it is still a challenge to identify “dark matter”. Some steps might be helpful to deal with the dark matter. In addition to aligning individual sequence reads to database by researchers, contig analysis together with gene types cataloguing, and qualifying matching to viral gene families can identify viral metagenomic sequence more easily [2].

### 2.3. Composition of the Human Virome

**Figure 2:**
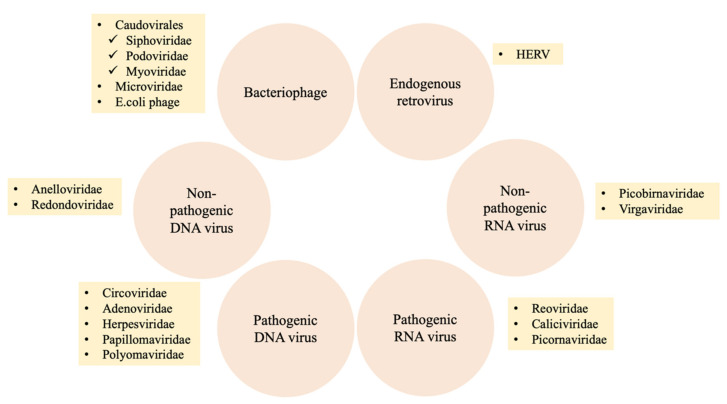
Composition of the human virome. Bacteriophages, eukaryotic RNA viruses, eukaryotic DNA viruses, and endogenous retroviruses colonize the human body. The virome, which includes bacteriophages from *Caudovirales* and *Microviridae* and DNA viruses such as those in *Anelloviridae*, is related to the pathogenesis of diseases. Abbreviation: *E. coli*, *Escherichia coli*; HERV, Human endogenous retroviruses.

The human virome comprises the set of all viruses in the human body, including bacteriophages, eukaryotic viruses, and endogenous retroviruses (Figure 2) [15,22]. These viruses are present throughout the human body, in the gut, skin, and oral cavity, and can be found in various sample types, including blood, feces, and cerebrospinal fluid. Certain viruses can be acquired through birth and continue to be seeded from the maternal microbiome and shaped by dietary habits as well as intimate contact [12].

Bacteriophages are viruses that infect bacteria and consist of infectious particles with at least two components, i.e., nucleic acid and protein [23]. There are four types of interactions for phages to engage. First, phages in lytic phase can infect cells, produce viral molecules, assemble particles and lead to host cells lysis. Second, phages can inject their genomes into bacterial cell, and the genomes may integrate into the bacterial chromosome in lysogenic growth. Another two special interactions are prophage and pseudolysogeny. The prophage stage would remain until a suitable induction signal is detected, phage genome would then move on to direct lytic growth. In pseudolysogeny, phage genome cannot actively direct lytic growth, as there is a loose interaction when the phage genome is in the bacterial cell [24,25]. *Caudovirales*, including *Myoviridae*, *Podoviridae*, *Siphoviridae* and members of the family *Microviridae* are commonly predominant in the human gastrointestinal tract [2]. Moreover, *Caudovirales* are commonly found in the oral cavity and respiratory tract [2].

Eukaryotic viruses that infect eukaryotic cells include both DNA and RNA viruses. Eukaryotic DNA viruses in the human gut include single- and double-stranded DNA viruses. Members of *Anelloviridae* and *Circoviridae* are single-stranded DNA viruses. Double-stranded DNA viruses, such as those in *Adenoviridae*, *Herpesviridae*, *Papillomaviridae*, and *Polyomaviridae*, are commonly related to infectious diseases [2,26,27]. Anelloviruses are the most common eukaryotic DNA viruses in the human oral cavity and respiratory tract followed by members of *Redondoviridae* [2]. On the other hand, eukaryotic RNA viruses are more commonly detected in the human gut virome and are divided into pathogenic and nonpathogenic viruses. Nonpathogenic RNA viruses include those in *Picobirnaviridae* and *Virgaviridae*, plant viruses that appear to originate from food. *Reoviridae* (such as rotavirus), *Caliciviridae* (such as norovirus), and *Picornaviridae* (such as enterovirus) contain pathogenic RNA viruses that are commonly associated with gastroenteritis [2,27].

Human endogenous retroviruses (HERVs) are fossil viruses that account for approximately 8% of the human genome. HERVs also contain proviral DNA and integrated genomes [28]. HERVs can integrate into the human genome, and the proviral DNA in germline cells can be transmitted to offspring. HERV proteins and replication of HERVs are associated with several diseases, including autoimmune, neurodegenerative, and chronic inflammatory diseases [15]. For example, envelope proteins from HERV-W and HERV-K are involved in the pathophysiological pathways of multiple sclerosis and amyotrophic lateral sclerosis [28].

## 3. The Virome and Human Disease

**Figure 3:**
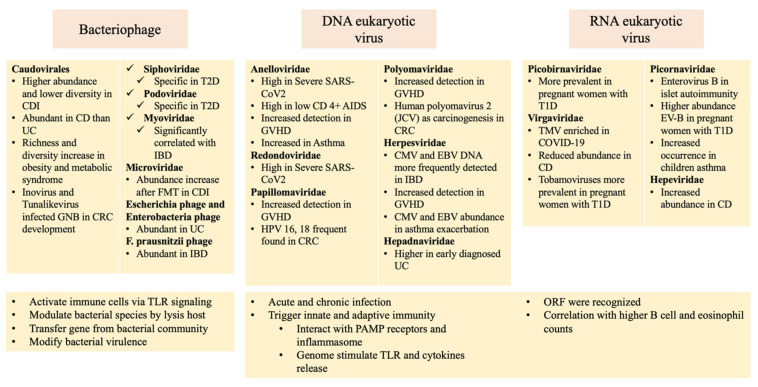
Alteration of the human virome in diseases and possible pathogenic mechanisms. Changes to bacteriophages, eukaryotic DNA viruses, and eukaryotic RNA viruses are shown in yellow squares. Pathogenetic pathways associated with bacteriophages and eukaryotic viruses are illustrated below the yellow squares. Abbreviations: AIDS, acquired immunodeficiency syndrome; CD, Crohn’s disease; CDI, *Clostridioides difficile* infection; CMV, cytomegalovirus; COVID-19, coronavirus disease 2019; CRC, colorectal cancer; EBV, Epstein–Barr virus; EV-B, enterovirus type B; FMT, fecal microbiota transplantation; GVHD, graft-versus-host disease; IBD, inflammatory bowel disease; SARS-CoV-2, severe acute respiratory syndrome coronavirus 2; T1D, type 1 diabetes; T2D, type 2 diabetes; UC, ulcerative colitis; TLR, Toll-like receptor; PAMP, pathogen associated molecular pattern; ORF, open reading frames.

Microbiome dysbiosis, including alteration of the virome, occurs at several sites in the human body. Using novel metagenomic approaches, the potential roles of the human virome have been studied in not only maintenance of homeostasis but also disease promotion. Although several direct causal relationships remain to be elucidated, we present a summary of current evidence for associations between the virome and human diseases, including SARS-CoV-2 infection, human immunodeficiency virus (HIV) infection, *Clostridioides difficile* infection (CDI), inflammatory bowel disease (IBD), graft-versus-host disease (GVHD), type 1 diabetes (T1D), type 2 diabetes (T2D), obesity, hypertension, asthma, chronic obstructive pulmonary disease (COPD), and colorectal cancer (CRC), in Table 1 and Table 2 and Figure 3.

### 3.1. Infectious and Inflammatory Diseases

#### 3.1.1. Severe Acute Respiratory Syndrome Coronavirus 2

The novel virus SARS-CoV-2 is responsible for the COVID-19 pandemic [70]. The gut microbiome has been shown to be a risk factor for COVID-19 [71], and dysbiosis was also seen in patients [72]. A mouse model study showed that the gut microbiome was affected by SARS-CoV-2 infection, with the microbiome composition associated with disease severity and recovery processes [73]. A case-control study showed that pepper mild mottle virus (RNA virus) originating from the diet was underrepresented in COVID-19 patients. The majority of DNA viruses enriched in the feces of non-COVID-19 controls were bacteriophages. On the other hand, COVID-19 patients exhibited enrichment for eukaryotic viruses, and *Escherichia* and *Enterobacter* phages were also prominent. An increase in phage abundance was associated with gut inflammation and the host interferon response [29]. Increased stress-, inflammation-, and virulence-associated gene expression was observed, suggesting that these viruses play a role in the host immune response to SARS-CoV-2 infection [29]. Moreover, after disease resolution, delayed SARS-CoV-2 viral shedding and persistent gut virome dysbiosis were noted [74]. Immune dysregulation driven by SARS-CoV-2 infection may also promote imbalance of the microbial and viral ecosystems of the human body that could result in long COVID or post-acute sequelae of COVID-19 [70].

After the gut microbiome, the human oral microbiome is the second largest microbial community. A cross-sectional study demonstrated oral dysbiosis in COVID-19 patients compared to matched controls that was significantly correlated with symptom severity and increased local inflammation. In addition, a decreased mucosal sIgA response was observed in patients with more severe symptoms. Therefore, the human oral microbiome profile is associated with susceptibility to SARS-CoV-2 infection, facilitation of inflammation, virus replication, and/or a protective IgA response [30]. Moreover, with regard to the respiratory virome, the results of sequencing analysis by Kim et al. demonstrated an 8% coinfection rate with rhinovirus or influenza virus [75]. One case–control study reported severe microbiota dysbiosis with enrichment of tobacco mosaic virus (TMV) in the COVID-19 group. The significant correlation between TMV and SARS-CoV-2 implies marked inflammatory interactions between the host, SARS-CoV-2, and other microbes in the lungs [31]. Another case–control study also demonstrated upper respiratory microbiome dysbiosis in COVID-19 patients, and greater change over time than in critically ill patients without COVID-19. Microbiome diversity was inversely correlated with disease severity, and the microbiome composition was associated with the lymphocyte/neutrophil ratio and the peripheral blood mononuclear cell profile in blood. Viruses from *Anelloviridae* and *Redondoviridae* were shown to be more frequent colonizers and had higher titers in severe disease. Therefore, the respiratory tract microbiome and commensal viruses are disturbed in COVID-19 and are correlated with systemic immune parameters [32].

#### 3.1.2. Human Immunodeficiency Virus

Immunodeficiency due to HIV infection has been shown to be associated with alterations in the enteric virome and bacterial microbiome, which may contribute to acquired immunodeficiency syndrome (AIDS) disease progression [76]. A plasma virome study showed that nucleic acids from hepatitis B virus (HBV), hepatitis C virus (HCV), and pegivirus A (GBV-C) were detected in some HIV-infected patients receiving antiretroviral therapy (ART), and anellovirus DNA was detected in HIV-suppressed subjects [34]. Moreover, higher levels of T-cell activation were not correlated with specific anellovirus genotypes [34]. Another study compared HIV-infected subjects with low versus high CD4^+^ T-cell counts and found that a higher anellovirus level was observed in patients with lower CD4^+^ counts [33]. The proportion of HERV reads was increased in AIDS patients with lower CD4^+^ counts than in HIV-infected subjects with high CD4^+^ counts. Therefore, the progression of AIDS is associated with changes in the plasma concentrations of commensal viruses [33].

In addition to members of the plasma virome, cervical swab samples from HIV/human papillomavirus (HPV)-co-infected women showed the presence of four viral families, *Papillomaviridae*, *Anelloviridae*, *Genomoviridae*, and *Herpesviridae.* Papillomaviruses were more abundant in women with premalignant cervical lesions. The anellovirus read abundance was inversely correlated with the host CD4^+^ T-cell count. Women with high rates of genomovirus or herpesvirus reads had increased risk of hosting a vaginal microbiome with a high proportion of anaerobic bacteria [35]. Another study analyzed semen samples from men with HIV. Anelloviruses, cytomegaloviruses (CMVs), and several genotypes of HPVs were detected. Viruses were more frequently shed by individuals with detectable HIV viremia. Individuals not on ART or those with lower CD4^+^ T cell counts tend to exhibit increased shedding. Therefore, control of viremia through ART may lower the shedding of other viruses in semen [36].

#### 3.1.3. Clostridioides Difficile Infection

Subjects with CDI were shown to have a significantly greater abundance of bacteriophages in *Caudovirales* and lower *Caudovirales* diversity, richness, and evenness compared with healthy controls from the same household [37]. Significant correlations were observed between the bacterial families *Proteobacteria*, *Actinobacteria*, and *Caudovirales* taxa in CDI [37]. A significant decrease in the abundance of *Caudovirales* taxa were observed in patients with CDI after FMT. The relative abundance of *Microviridae* in recipients after FMT increased significantly in comparison to before transplantation [38]. Another study showed that FMT recipients established a virome distinct from the donor that included sequences of algal giant viruses (chloroviruses), double-stranded DNA viruses present in inland aqueous environments [77].

#### 3.1.4. Inflammatory Bowel Disease

Intestinal dysbiosis is one of the causes underlying the pathogenesis of IBD, including ulcerative colitis (UC) and Crohn’s disease (CD) [78]. One study showed that *Faecalibacterium prausnitzii* was generally depleted in IBD patients. Phages of *F. prausnitzii* were more abundant in IBD patients compared to healthy controls, suggesting that *F. prausnitzii* phages may play a role in the disease [44]. Another study showed that the bacterial composition in new-onset patients with CD differed from that in the controls, and the bacterial community reflected the disease status of individuals more accurately than their viral counterparts. Moreover, the abundance of phages that infect *Clostridiales, Alteromonadales,* and *Clostridium acetobutylicum*, as well as that of members of the *Retroviridae* family were increased in IBD patients compared with healthy subjects [41].

A shift from a stable core of virulent bacteriophages to temperate phages may be associated with IBD [45]. One study demonstrated the greatest abundance of *Caudovirales* members in CD ileum samples and CD gut wash samples, whereas only one phage sequence was detected in colonic samples, suggesting that the bacterial species associated with ileal CD may have a predisposition toward phage infection [39]. Another study reported a significant expansion of *Caudovirales* bacteriophages in both CD and UC; however, no increases in *Microviridae* richness or diversity were found [40]. In another study, *Caudovirales* phages were more abundant in CD than in UC. The richness of viral strains in *Microviridae* was higher in controls than in CD patients [46]. Moreover, inverse correlations were observed between *Caudovirales* diversity and bacterial richness and diversity, suggesting that the bacteriophage expansion was not simply the result of increases in the populations of their bacterial hosts [40]. *Myoviridae* was shown to be significantly correlated with IBD, whereas *Microviridae* and crAss-like phages were significantly correlated with healthy controls. Moreover, there was also no significant separation of virome composition between a UC flare or remission [45]. Expansion of *Caudovirales* bacteriophages was observed in the UC group. Escherichia and enterobacteria phages were more abundant in the mucosa of UC patients than in that of the controls. The trans-kingdom correlations between mucosa viruses and bacteria were also significantly depleted in UC [48]. Additionally, patients with very-early onset IBD (VEO-IBD) had a higher ratio of *Caudovirales* to *Microviridae* compared with healthy controls. An increase in the level of *Caudovirales* was associated with immunosuppressive therapy. *Anelloviridae*, which was also positively associated with immunosuppressive treatment, was more prevalent in VEO-IBD patients compared to healthy controls, suggesting that *Anelloviridae* DNA may be useful for monitoring the effectiveness of immunosuppression [49].

Several studies of eukaryotic viruses and endogenous retroviruses have demonstrated the diversity and abundance of HERVs among IBD colon samples and suggested that infection with *Herpesviridae* such as Epstein-Barr virus (EBV) may trigger the expression of HERVs in the colon [42]. CMV and EBV DNA were detected more frequently in the mucosa of patients with IBD compared with controls. The EBV viral load was similar in inflamed and non-inflamed mucosa, unaffected by therapeutic regimens, and not correlated with disease activity, suggesting that EBV may be involved in the onset of IBD rather than in its clinical evolution [43]. Early diagnosed UC patients exhibited significantly higher levels of *Hepadnaviridae* transcripts in comparison with controls and lower levels of *Polydnaviridae* and *Tymoviridae* in the intestinal mucosa. Moreover, CD patients exhibited increased abundance of *Hepeviridae* with a reduced abundance of *Virgaviridae* in the mucosa compared to controls [47].

#### 3.1.5. Graft-Versus-Host Disease

A study exploring virome dynamics in allogeneic hematopoietic stem cell transplantation (HSCT) and enteric GVHD showed increased rates of detection and numbers of sequences of persistent DNA viruses, such as anelloviruses, herpesviruses, papillomaviruses, and polyomaviruses in individuals with enteric GVHD, and these findings were also accompanied by reduced phage richness. Picobirnavirus was predictive of the occurrence of severe enteric GVHD and correlated with higher fecal levels of two GVHD severity markers, calprotectin and α1-antitrypsin, suggesting an unexpected association of picobirnavirus with early post-transplant GVHD [50].

### 3.2. Chronic Diseases

#### 3.2.1. Type 1 Diabetes

A complex interplay of genetic predisposition and environmental exposures resulted in the pathogenesis of T1D. Accumulating evidence supports the influence of environmental factors, particularly bacteria and viruses, in the etiology of T1D [79]. A review of studies in animal models indicated that alterations in gut bacterial composition precede disease onset [80] and short-chain fatty acids other than butyrate produced by gut bacteria were shown to be elevated in T1D patients, thus suggesting a causal role of the gut microbiome in islet destruction [81]. Moreover, autoimmune destruction of β-cells upon viral infection may lead to insulin deficiency. Viral infection was shown to further increase activated autoreactive T cells, leading to autoimmune disease [82].

Another case–control study collected fecal samples and found changes in the eukaryotic virome, with *Circoviridae*-related sequences associated with protective effects against autoimmunity. Changes in bacteriophages may be associated with autoimmunity, and higher Shannon diversity of *Podoviridae* and richness of *Myoviridae* were found in controls in comparison to T1D patients; however, the relationships between bacteriophages and the development of autoimmunity in T1D remain to be studied further [51]. Analysis of a public longitudinal fecal microbiome dataset revealed an increase in the *Escherichia coli* phage*/E. coli* ratio prior to *E. coli* depletion in children who had developed T1D, suggesting that the decrease in *E. coli* was due to prophage activation, with phage-mediated lysis of *E. coli* leading to the release of amyloid aggregates into *E. coli* biofilms [52]. A nested matched case–control study suggested that prolonged enterovirus B (EV-B) infection may be involved in the development of islet autoimmunity but not T1D. Furthermore, fewer early-life human mastadenovirus C infections and the rs6517774 variant of the coxsackie and adenovirus receptor gene were independently correlated with islet autoimmunity [53]. Another study characterized the gut virome of pregnant women with and without T1D. Eukaryotic viruses were shown to be prevalent in the gut of pregnant women. Two viruses, picobirnaviruses and tobamoviruses, were more prevalent in pregnant women with T1D than in nondiabetic controls. Three EV-Bs (CVB4, CVB3, and ECHOvirus E18) were present at greater abundance in women with T1D in comparison to nondiabetic controls. By contrast, four EV-As (CVA10, CVA16, CVA5, and CVA14) were more abundant in pregnant women without T1D [54]. Another study in African and Asian countries showed no differences in the frequency of eukaryotic virus species or genera between children with T1D and nondiabetic controls. However, there were more frequent HERV signals in T1D, which requires further exploration [55]. A recent meta-analysis included case–control studies examining the virome using mNGS in children who had developed islet autoimmunity/T1D; this study identified weak but significant associations between islet autoimmunity and the number of stool samples positive for all enteroviruses as well as the number of stool samples positive specifically for EV-B [79]. Although significant differences were reported in the gut virome of T1D patients compared to nondiabetic controls, the sample sizes were very small [83].

#### 3.2.2. Type 2 Diabetes and Obesity

T2D is a metabolic disease that results from obesity-linked insulin resistance, and several studies have demonstrated associations with compositional changes in the gut microbiota [84]. Recent studies also showed that diabetic retinopathy, a complication of T2D, is related to dysbiosis of the gut microbiome [85]. A case–control study analyzed metagenomic sequencing data from fecal samples from both T2D patients and healthy adult controls. A significantly increased gut phages was found in the T2D group, and seven phage operational taxonomic units (pOTUs), consisting of four *Siphoviridae*, two *Podoviridae*, and one unclassified family, were significantly specific to T2D. The inferred bacterial hosts of these pOTUs were enterobacteria, *Escherichia*, *Lactobacillus*, *Pseudomonas*, and *Staphylococcus*. A complex core interaction was identified between bacteria and phages in the human gut ecosystem, suggesting that significant alterations to the gut phageome cannot be explained simply by co-variation with altered bacterial hosts [56].

Obesity is a global health problem that has negative impacts on quality of life. A number of studies have suggested a relation between obesity and disruption of normal microbiome composition [59]. An animal study showed that feces from obese mice contain greater viral contents in terms of total viral DNA and RNA than did those from normal controls; this increase was strongly correlated with metabolic measures, such as body weight, fat mass, and fasting blood glucose level. Total viral content was positively correlated with Firmicutes and negatively correlated with Bacteroidetes and *Bifidobacterium* [86]. In the gut dsDNA virome derived from fecal samples of school-aged children, the bacteriophages mainly belonged to *Caudovirales*. Phage richness and Shannon diversity tend to increase in individuals with obesity and metabolic syndrome. The abundance of several phage contigs was correlated with gut bacterial taxa and anthropometric and biochemical parameters, such as a high body mass index and high triglyceride and glucose levels in individuals with obesity and metabolic syndrome [57]. Another study of serum samples from adults and children in Qatar showed that obese subjects had higher herpes simplex virus 1 (HSV-1) seropositivity and seroprevalence than did lean adults. Higher prevalence of antibodies against several peptide epitopes of HSV-1/2 is positively associated with obesity, suggesting that viral peptides may play a role in adipogenesis [58]. Patients with obesity and T2D had decreased gut viral richness and diversity compared with lean controls. Eleven viruses, including *Escherichia* phages, *Geobacillus* phages, and *Lactobacillus* phages, were enriched in obese subjects. The extensive trans-kingdom correlations between viruses and bacteria observed in lean controls were significantly decreased in subjects with obesity and T2D [60]. A study comparing the gut virome of obese subjects before and after treatment showed that the virome composition changed after obesity intervention. A lower alpha diversity index for the gut virome was found in the obese subjects prior to treatment compared to after treatment. Only four viruses were identified in the core virome prior to treatment, whereas post-treatment, at least 13 viruses shaped the core virome and resulted in higher core diversity of DNA viruses [59]. With regard to fatty liver disease, non-alcoholic fatty liver disease (NAFLD) patients with advanced liver cirrhosis exhibited a significant decrease in intestinal viral diversity compared to those with a low level of cirrhosis or healthy controls. Severe NAFLD patients exhibited a significant reduction in the proportion of bacteriophages compared with other intestinal viruses. The study also developed a model including a viral diversity index and simple clinical variables that accurately identified patients with severe NAFLD and fibrosis [63].

#### 3.2.3. Hypertension

Gut microbiota dysbiosis has been observed in relation to hypertension, including decreased diversity, altered microbial structure, compositional changes in taxa, and alterations of microbial function and nutritional and microbial interactions [87]. One study compared the gut viromes of pre-hypertensive and hypertensive patients. Analyses with viruses may be superior to those relying on bacteria in terms of resolution and discriminatory power for distinguishing samples from healthy individuals and those with pre-hypertension or hypertension. The pervasiveness of virus–bacteria linkages increased in the following order: healthy individuals, those with pre-hypertension, and those with hypertension [61]. Pathways for the synthesis of arginine, proline, and ornithine were shown to be increased in pulmonary arterial hypertension (PAH). In addition, groups of bacterial communities associated with trimethylamine/trimethylamine *N*-oxide and purine metabolism were increased in PAH. Virome analysis also showed the enrichment of enterococcal phages and the relative depletion of lactococcal phages in PAH [62].

#### 3.2.4. Asthma and Chronic Obstructive Pulmonary Disease

With regard to respiratory diseases, a recent review found that respiratory syncytial virus (RSV) is a risk factor for respiratory morbidities and could worsen the symptoms of asthma [88]. In addition, rhinoviruses (RVs) have been linked to asthma exacerbation due to their action on mucin hypersecretion [89]. A study of children with asthma or pneumonia showed that RV-C, bocavirus 1, RSV-B, and parvovirus B19 were more prevalent in the asthma group, whereas bacteriophage EJ-1, torque teno mini virus, *Streptococcus* phage, RSV-B, and RV-A were more prevalent in the pneumonia group, and torque teno virus (TTV) was found in both groups with a similar number of reads [64]. Another study of asthma showed that dysbiosis of the nasopharyngeal virome was correlated with the severity of asthma. The major components of dysbiosis were bacteriophage deficiency and increased eukaryotic viruses, especially anelloviruses. Decreased connectivity within the virome-related microbiome, a decreased number of bacteriophage–bacterium pairs, and increased cross-family occurrence of picornaviruses within the virome were found in children with asthma [65]. Another study showed that CMV and EBV were more abundant in patients with asthma who experienced exacerbation, and the abundance was correlated with more severe asthma, a lower asthma control test (ACT) score, and reduced lung function. By contrast, bacteriophages were severely reduced in patients with asthma, with the decrease significantly and positively correlated with the ACT score and forced expiratory volume in 1 s/forced vital capacity ratio [66]. In another study of COPD, the viral pathogens detected using mNGS were HSV type 1 and coronavirus OC43. COPD patients with viral pathogens had lower percentages of bacteriophages, suggesting skewing of the virome during infection, with potential consequences for the bacterial populations [67].

### 3.3. Cancer

Viruses have been demonstrated to account for 10–15% causative agents of all cancers. Several DNA viruses, including Kaposi’s sarcoma herpesvirus, Merkel cell polyomavirus, EBV, HPV, HBV, and simian virus 40, as well as at least two RNA viruses, human T-lymphotropic virus-1 and HCV, have been shown to be associated with carcinogenesis [90]. Direct transformation is one mechanism of viral carcinogenesis in which the virus expresses viral oncogenes that can directly transform infected cells. Conversely, virus-induced chronic infection and inflammation can also function as indirect mechanisms of transformation [91].

#### Colorectal Cancer

CRC is one of the most common cancers, ranking third in incidence and second in mortality among all cancers worldwide. The gut microbiota was found to be involved in CRC formation, progression, and its response to treatment [92].

One meta-analysis showed that HPV 18 was found more frequently in CRC patients from Asia and Europe, whereas HPV 16 was more prevalent in colorectal tumors in South American patients [93,94]. Several studies also showed the increased risk of developing CRC in HPV-infected patients [95,96]. MYC, WNT-5A, and AXIN2 were shown to be upregulated in HPV-positive CRC tissues compared to HPV-negative tissues, suggesting a possible association between HPV infection and the development of CRC [97]. Moreover, human CMV is also often found in CRC patients with poor prognosis [98,99,100,101]. Components of the Toll-like receptor (TLR) 2 pathway, such as TLR4, NF-κB, and TNF-α [102], as well as the Wnt signaling pathway, which is associated with cell proliferation and migration, were upregulated in CRC cells infected with CMV [103]. Induction of Bcl-2 and cyclo-oxygenase-2 proteins, which are related to the progression of colon cancer, was observed in CRC cells with CMV infection [104]. Furthermore, human polyomavirus 2 (also known as JCV) was reported to possibly play a role in the carcinogenesis of CRC [105]. One meta-analysis reported JCV as an oncogene virus that could increase the likelihood of CRC [106]. *T-antigen* (*T-Ag*), a transforming gene encoded by JCV, is involved in oncogenesis by inducing the methylation of tumor suppressor gene promoters [107,108]. Deregulation of the Wnt signaling pathway through β-catenin was also shown to be mediated by *T-Ag* [109].

One study of CRC built a model to illustrate the relations between the virome, bacterial community, and CRC. Alteration of the bacterial community by bacteriophages, including those from *Siphoviridae* and *Myoviridae* and unclassified phages, allowed colonization by driver bacteria such as *Fusobacterium*. Passenger bacteria then facilitate epithelial cell transformation and bacterial infiltration, producing carcinogenic reactive oxygen species (ROS) and polyspermines [68,110]. The enrichment of *Orthobunyavirus* sequences was found in CRC patients. On the other hand, the enrichment of *Inovirus* and *Tunalikevirus*, which infect Gram-negative bacteria, including bft-positive enterotoxigenic *Bacteroides fragilis*, *Fusobacterium*, and pks-positive genotoxic *E. coli*, may represent a trans-kingdom microbial interaction resulting in the development of CRC. A combination of four taxonomic markers—*Betabaculovirus*, *Epsilon15likevirus*, *Mulikevirus*, and *Punalikevirus*—was shown to be associated with the reduced survival of patients with CRC [69].

### 3.4. Possible Pathogenic Relations between the Human Virome and Disease

Interactions between phages, eukaryotic viruses, bacteria, and the host immune system likely play important roles in host immune homeostasis. Eukaryotic viruses can cause acute or chronic infection, but they can also protect the host by triggering innate or adaptive immunity. Phages produced by bacteria can be taken up by immune cells and activate immune responses via TLR signaling. Bacteriophages can also change the abundance of bacterial species by lysing their bacterial hosts, modifying bacterial virulence, and inducing bacterial phagocytosis [2,111,112,113]. Here, we summarize virome interactions in human diseases, as shown in Figure 3.

Virome interactions with respiratory diseases are strongly associated with *Anelloviridae* and mediated by TLR signaling. Anelloviruses influence not only innate immunity but also adaptive immunity [114]. TTV, the best-studied member of the *Anelloviridae*, may interact with pathogen-associated molecular pattern (PAMP) receptors and activate the inflammasome. The TTV genome and specific CpG motifs stimulate immune cells via TLR9 [115]. The triggering of inflammatory cytokines, including IFN-α, IL-6, and IL-12, would depend on the number and/or types of nucleotides flanking the CpGs. Moreover, the ORF2 protein of TTV influences the activity of NF-κB, which in turn activates the transcription of genes such as those encoding IL-6, IL-8, and cyclo-oxygenase-2. Increased TTV load was also shown to be associated with lower T lymphocyte counts but higher B cell and eosinophil counts [114,116], and virus infection can lead to the local release of type 1 IFN. The upregulation of FcεR1 expression on airway mucosal dendritic cells would result in the increased recruitment and local activation of T-helper type 2 (Th2) cell effectors as well as the subsequent expansion of Th2 memory cell clones [117].

Phages alter the bacterial microbiome and play a role in intestinal diseases. *Caudovirales* phages are closely related to immune response stimulation and the aggravation of colitis. For instance, they can facilitate horizontal gene transfer from bacterial communities, including genes related to pathogenesis and antibiotic resistance. Additionally, lysis of bacterial hosts by these phages would alter the abundance of specific gut bacterial species [118]. Moreover, nucleic acids released by lysis of bacteria would act as PAMPs and antigens to trigger inflammatory signaling. A recent study reported that phages produced by pathogenic bacteria can be taken up by antigen-presenting cells in mice to induce type I IFN responses via TLR3- and TRIF-dependent viral pattern recognition receptors. Type 1 IFN inhibits TNF production and limits bacterial phagocytosis, which may result in impaired bacterial clearance and more frequent infection [119]. Another study reported the expansion of CD8^+^ and IFN-γ-producing CD4^+^ T cells in the mucosa of mice fed *E. coli* phages isolated from the human gut [120]. Furthermore, this study also showed that *Lactobacillus*, *Escherichia*, and *Bacteroides* bacteriophages and phage DNA stimulated IFN-γ via the nucleotide-sensing receptor TLR9 [120]. In addition to bacteriophages, the bacterial community has been shown to promote eukaryotic virus replication and pathogenesis. Poliovirus was shown to bind to bacterial lipopolysaccharide, thus enhancing virion stability, suggesting that microbiota-mediated stabilization promotes the fitness of poliovirus in the environment [121]. Mouse mammary tumor virus, a member of *Retroviridae*, also binds to lipopolysaccharide and induces host TLR4 signaling and the immunosuppressive cytokine IL-10, thus evading host immunity and augmenting transmission [122]. These trans-kingdom interactions between eukaryotic viruses, bacteriophages, bacteria, and hosts may impact human health and disease.

## 4. Therapeutic Applications

### 4.1. Fecal Microbiota Transplantation

The efficacy of FMT may be related to the transfer of bacteriophages and viruses in CDI patients. Phage communities of recipients were shown to be similar in composition, diversity, and richness to those of donors, suggesting that enteric phages are transferred from the donor to the recipient during FMT [123]. A recent study demonstrated that colonization by donor *Caudovirales* bacteriophages was correlated with the treatment efficacy of FMT in CDI patients [37]. *Microviridae* counts in recipients after FMT were also shown to be correlated with the therapeutic efficacy of FMT [38]. Another study showed that the transfer of fecal filtrate containing bacterial components, metabolites, and bacteriophages that contribute to the normal intestinal microenvironment rather than FMT was sufficient to restore normal stool habits and eliminate symptoms in CDI patients [124], suggesting that the gut virome of FMT donors should be considered in future.

In addition to CDI, the efficacy of this type of treatment has also been examined in irritable bowel syndrome, GVHD, and T2D. A systematic review and meta-analysis assessing FMT as treatment for active UC reported higher rates of clinical and endoscopic remission and no statistically significant increase in adverse events compared to placebo-treated controls [125]. Low eukaryotic viral richness is a novel diagnostic marker for treatment response due to the association with FMT success in patients with UC [126]. Moreover, FMT was shown to have potential for treating severe colitis associated with GVHD following HSCT. After serial FMTs, the compositions of the gut bacteriome, mycobiome, and virome differed with a stable rise in diversity. The abundance of TTV decreased after FMT, and the relative abundance of *Caudovirales* bacteriophages increased [127]. In another recent animal study, fecal virome transplantation (FVT) from lean mice reduced weight gain and normalized glucose tolerance in obese recipients. These findings demonstrate the efficacy of FVT in obesity and T2D [128]. A randomized clinical trial also showed that FMT could enhance the level and duration of microbiota engraftment in obese patients with T2D. A combination of lifestyle interventions with FMT was reported to lead to improvements in the lipid profile and liver stiffness of obese recipients with T2D [129].

A review summarized several approaches involving modification of the gut microbiota to prevent CRC and improve treatment responses, including dietary intervention, the use of prebiotics and probiotics, and FMT [92]. Another review also reported evidence of beneficial effects of microbiome modulation in cancer and summarized the results of trials that applied FMT in cancer patients undergoing immunotherapy, chemotherapy, radiation, or targeted therapy [130]. Furthermore, FMT may potentially help with overcoming immunotherapy resistance and ameliorating adverse effects in CRC. Phage transfer may also increase the response to immunotherapy by inducing T-cell cross-reactivity with cancer antigens [131]. Finally, immune checkpoint inhibitor-associated colitis was successfully treated with FMT, as seen by reconstitution of the gut microbiome and a relative increase in the proportion of regulatory T cells within the colonic mucosa [132].

With regard to donor and recipient selection for FMT, as the bidirectional gut–lung axis during COVID-19 infection can directly (via ACE2 receptors and gut microbial metabolites) and indirectly (via the immune system) affect the gut and lung, FMT screening protocols are required during and after the COVID-19 pandemic [8]. Another review summarizing donor screening recommendations indicated that diabetes mellitus, prior cardiovascular events, and exposure in clinical healthcare settings should be considered exclusion criteria until more is known about the associations of these conditions with the human gut microbiome [133].

### 4.2. Phage-Based Therapy

Phage therapy could represent a therapeutic approach to restore intestinal eubiosis due to its immunomodulatory and bactericidal effects against bacterial pathogens. Moreover, modified temperate phages can suppress the transcription of bacterial virulence factors [134]. To examine the effects of phage therapy on the gut microbiota and systemic inflammatory markers in a healthy human population, Febvre et al. used a commercial cocktail of *E. coli*-targeting bacteriophages and showed that after consuming phages, there was no global disruption of the microbiota and no alterations to inflammatory markers or lipid metabolism [135].

*Clostridioides difficile* and adherent invasive *E. coli* (AIEC) may play roles in the pathogenesis of IBD, whereas *Fusobacterium nucleatum* may be related to the pathogenesis of CRC, and all have been studied as targets of lytic phages. One animal study that assessed bacteriophages targeting the prototype AIEC strain, LF82, demonstrated the reduction of ileal and colonic colonization by AIEC and symptoms of colitis in mice [136]. Another study revealed marked downregulation of genes associated with CRC, tumor growth, metastasis, and invasion of gastrointestinal cancer in animals treated with *E. coli* bacteriophages [120]. Yet another study showed that azide-modified phages that inhibit *F. nucleatum* significantly augmented the efficacy of first-line chemotherapy treatment of CRC without severe adverse effects. Therefore, the application of phages to modulate the gut microbiota may be a new approach for treating CRC [137].

Inhaled phage therapy has the potential to transform the prevention and treatment of bacterial respiratory infections, including those caused by antibiotic-resistant bacteria [138]. Several studies showed that it has great potential for managing these difficult-to-treat bacterial infections in severely ill COVID-19 patients [139]. Moreover, by inhibiting the activation of NF-κB and ROS production, phages were shown to downregulate excessive inflammatory reactions in COVID-19 patients [140].

Despite the potential therapeutic effects of phage therapy, some issues remain to be resolved. For example, it remains to be determined whether the lytic or lysogenic life cycle is more suitable for the destruction of harmful bacterial strains and treatment of related diseases, and how the immune system reacts to various phage-associated antigens both systemically and locally [141]. Moreover, there is still a lack of standardization and legal frameworks for establishing regulatory and safety protocols for the clinical application of phages [134].

### 4.3. Oncolytic Therapy

Cancer virotherapy based on oncolytic viruses (OVs) can modulate the tumor microenvironment to reverse the immunosuppressive state and subsequently stimulate antitumor immunity. In addition, these viruses can be designed to target cancer cells without damaging normal cells. Various OVs have been developed for anticancer therapy, including those based on HSV, vaccinia virus, adenovirus, reovirus, and measles virus. Ongoing clinical trials of this type of therapy in CRC patients have been summarized in a review article by Wang et al. [16].

A wide variety of OVs are currently undergoing phase I/II clinical trials, with 130 clinical trials in this field registered on ClinicalTrials.gov (accessed on 20 January 2022) and more than 40 such trials completed to date. Anticancer viral cocktails combining engineered OVs and bacteriophages would have the benefits of both oncolytic targeting of cancerous cells and promotion of anticancer immune responses [142]. However, it is necessary to be aware of safety issues, particularly the possible adverse effects arising from this therapy. Further studies regarding the selection of optimal OVs and the safety of combining OV therapy with chemotherapy, radiotherapy, or immunotherapy are needed to establish clinical protocols [143].

## 5. Conclusions

Advances in technology and updating of viral genome databases have accelerated the pace of virus identification, resulting in increased interest in the human virome. Viral metagenomics involves a number of processes, including sample collection, storage, processing, sequencing, and bioinformatics analyses. Viral metagenomics may be subject to bias due to contamination, low abundance, variation in viral genomes, and a lack of standard annotation tools. Bacteriophages, eukaryotic viruses, and human endogenous retroviruses colonize the human body and interact with one another, triggering immune cells and activating immune responses. Bacteriophages can lyse host bacteria and alter both their abundance and virulence. Due to the role of the virome, FMT, which involves the transfer of bacteriophages and viruses from donors to recipients, exhibits efficacy in a number of diseases. Phage therapy, which modulates the immune system and results in bactericidal effects, has been shown to be efficacious in infectious and inflammatory diseases. However, clinical protocols for these therapies must be established after the COVID-19 pandemic.

Studies of the human virome and diseases have so far been limited to case–control series. The sample sizes of some of these studies are too small to determine the validity of the reported outcomes, and thus, they might have been subject to type II error. Moreover, some studies suffered from sampling bias, such as when the healthy controls were not typical of the population. The methods used for matching in case–control studies must also be taken into consideration, as the virome may be influenced by diet, geography, underlying diseases, and aging. Direct causal relations of viruses with disease and their pathogenic mechanisms cannot be determined through case–control studies and have been investigated in animal studies or in vitro experiments. However, these findings remain to be translated to humans.

The human virome is gradually being elucidated. A fuller understanding of the details of the mechanistic interactions between the virome and other components of the human microbiota will facilitate the development of novel therapeutic methods that target the lytic or lysogenic stages of viruses, provide phage-resistant probiotics to increase levels of probiotics influenced by certain phages, and include the design of vaccines against phage virions produced by pathogenic bacteria. Further translational and clinical studies are required to develop therapeutic approaches targeting the human virome to improve human health and wellbeing.

## Figures and Tables

**Table 1:** Human virome changes in infectious and inflammatory human diseases.

Human Disease	Samples	Case (*n*)	Control (*n*)	Virome Alternation	Reference
SARS-CoV-2	Feces	98 COVID-19 patients (3 asymptomatic, 53 mild, 34 moderate, 5 severe, 3 critical)	78 non-COVID-19 controls matched for gender and co-morbidities	More eukaryotic viruses, Escherichia phage and Enterobacter phage in COVID-19	[29]
SARS-CoV-2	Oral rinse samples	39 COVID-19 patients	36 healthy controls	Oral dysbiosis correlated with symptom severityA decreased mucosal sIgA response in severe COVID-19	[30]
SARS-CoV-2	Bronchoalveolar lavage (BAL) fluid	19 COVID-19 patients	23 healthy controls	Tobacco mosaic virus were enriched in the COVID-19	[31]
SARS-CoV-2	OP, NP swabs, endotracheal aspirates (ETA) and BAL	83 COVID-19 patients	42 healthy controls	*Anelloviridae* and *Redondoviridae* more frequent colonization in severe COVID-19.	[32]
HIV	Plasma	35 subjects were selected based on high or low CD4+ cell counts.		A higher level of Anelloviruses and HERV in subjects with lower CD4+ counts	[33]
HIV	Plasma	19 subjects under ART-mediated viral suppression		No association between Anellovirus DNA levels and the percentage of activated CD4 or CD8 T cells	[34]
HIV	Cervical sample	19 HIV/HPV co-infected women with multiple HPV infection		Papillomavirus reads more abundant in women with premalignant cervical lesions Anellovirus read abundance negatively correlated with host CD4+ T-cell counts.	[35]
HIV	Semen	42 men with HIV		Viruses more frequently shed by individuals with detectable HIV viremia	[36]
*C. difficile*	Feces	24 subjects with CDI	20 healthy controls	Higher abundance of bacteriophage *Caudovirales* and a lower diversity, richness in CDI.	[37]
Recurrent *C. difficile*	Feces	9 patients with recurrent *C. difficile* infection (3 or more confirmed episodes of CDI)		The relative abundance of *Microviridae* increased in the recipients after FMT, whereas *Caudovirales* decreased after FMT Most temperate phages behaved similarly to host bacteria in the FMTs	[38]
Crohn’s disease	Ileal and colonic biopsies Gut wash samples	6 CD patients (ileal biopsy) 6 CD patients (colonic biopsy) 3 CD patients (gut washes)	6 noninflammatory bowel disease patients (ileal biopsy)	*Caudovirales* family abundant in ileal and gut wash samples rather than colonic samples	[39]
Crohn’s disease Ulcerative colitis	Feces	18 subjects with CD 42 subjects with UC	12 household controls	Expansion of *Caudovirales;* however, no increases of *Microviridae* richness or diversity in both CD and UC An inverse correlation between *Caudovirales* and bacterial richness and diversity	[40]
Crohn’s disease	Feces and biopsy	20 patients with CD	20 healthy controls	Bacterial community reflects the disease status of individuals more accurately than their viral counterparts.	[41]
Crohn’s disease Ulcerative colitis	Surgical samples or colonoscopic biopsy	10 patients with IBD	5 subjects undergoing colonoscopy for colon cancer surveillance	Diversity and abundance of HERVs in IBD with *Herpesviridae* infection	[42]
Crohn’s disease Ulcerative colitis	Blood and mucosal samples	43 subjects with UC 52 subjects with CD	50 healthy subjects	CMV and EBV DNA more frequent in IBD Mucosal viral load not influenced by the therapeutic regimen	[43]
Crohn’s disease Ulcerative colitis	Feces	52 IBD patients	21 healthy controls	*F. prausnitzii* phages more prevalent or more abundant in IBD	[44]
Crohn’s disease Ulcerative colitis	Feces	27 subjects with CD 42 subjects with UC	61 healthy controls	Virulent core of bacteriophages dominant in healthy controls Temperate phages in IBD	[45]
Crohn’s disease Ulcerative colitis	Feces	7 children with CD 5 children with UC	12 similar aged controls	*Caudovirale* more abdundant in IBD The richness of viral strains in *Microviridae* higher in control	[46]
Crohn’s disease Ulcerative colitis	Ileum biopsy	55 colon-inflamed CD 161 ileum inflamed CD 61 colon-inflamed UC	42 healthy controls	Higher levels of *Hepadnaviridae* transcripts and lower levels of *Polydnaviridae* and *Tymoviridae* in UC Increased abundance of *Hepeviridae* with a reduced abundance of *Virgaviridae* in CD	[47]
Ulcerative colitis	Rectal mucosa	91 patients with UC	76 healthy controls	Expansion of *Caudovirales* bacteriophages in UC Escherichia phage and Enterobacteria phage more abundant in UC	[48]
Very early onset inflammatory bowel disease	Feces	54 subjects with VEO-IBD	23 healthy controls	Higher ratio of *Caudovirales* vs to *Microviridae* in VEO-IBD Higher level of *Anelloviridae* in VEO-IBD	[49]
GVHD	Feces	44 adults who underwent allogeneic hematopoietic stem cell transplantation		Increased numbers of sequences of persistent DNA viruses in enteric GVHD Picobirnaviruses were predictive of the occurrence of severe enteric GVHD	[50]

Abbreviation: SARS-CoV-2, severe acute respiratory syndrome coronavirus 2; COVID-19, coronavirus disease 2019; HIV, Human immunodeficiency virus; OP, oropharyngeal; NP, nasopharyngeal; BAL, bronchoalveolar lavage; HERV, Human endogenous retroviruses; ART, antiretroviral therapy; HPV, human papillomavirus; CDI, *C. difficile* infection; UC, Ulcerative colitis; CD, Crohn’s disease; GVHD, Graft-versus-host disease; CMV, Cytomegalovirus; EBV, Epstein–Barr virus; IBD, Inflammatory bowel disease.

**Table 2:** Human virome changes in human chronic diseases and cancer states.

Human Diseases	Samples	Cases (*n*)	Controls (*n*)	Virome Alternation	References
Type 1 DM	Feces	11 infants from Finland and Estonia based on HLA genotype	11 matched controls	Enrichment of *Circoviridae*-related sequences in control groups Higher Shannon diversity and richness of bacteriophages in control groups	[51]
Type 1 DM	Feces	10 children with autoantibodies (6 seroconverters and 4 children who developed T1D)	8 non-seroconverted HLA-matched controls	An increase of the E. coli phage/E. coli ratio in developed T1D	[52]
Islet autoimmunity/ Type 1 DM	Feces	323 islet autoimmunity and 95 T1D	418 controls	A prolonged enterovirus B (EV-B), Coxsackie and adenovirus receptor (CXADR), rs6517774 associated in islet autoimmunity	[53]
Type 1 DM (Pregnant women)	Feces	35 pregnant women with T1D	26 pregnant women without T1D	Picobirnaviruses and tobamoviruses more prevalent in pregnant women with T1D	[54]
Type 1 DM	Feces	73 children and adolescents shortly after T1D onset (Azerbaijan 19, Jordan 20, Nigeria 14, Sudan 20)	105 matched controls	More frequent endogenous retrovirus signal in T1D	[55]
Type 2 DM	Feces	71 T2D patients	74 non-diabetic Chinese adults	Increase number of gut phages in T2D	[56]
Obesity	Feces	10 Children with obesity 8 Children of obesity with metabolic syndrome	10 Children with healthy normal-weight	Phage richness and Shannon diversity increase in obese and metabolic syndrome	[57]
Obesity	Serum	273 obese Qatari adults 120 obese Qatari children	184 lean Qatari adults 111 lean Qatari children	Higher sero-prevalence of HSV1 and antibodies against several peptide epitopes of HSV-1/2 in obese	[58]
Obesity	Feces	21 pre-treated obese subjects	28 post-obesity-treated subjects: 2 exercise and diet, 17 Roux-en-Y gastric bypasses, 2 sleeve gastrectomies, 7 verticals banded gastroplasties	A lower alpha diversity index of the gut virome in pre-treated group Higher core diversity of DNA viruses in post-treated group	[59]
Obesity and Type 2 DM	Feces	128 obese subjects (BMI ≥ 28 kg/m^2^)	101 lean controls (BMI ≥ 18.5 and <23 kg/m^2^)	A decreased richness and diversity in obese Transkingdom correlations between viruses and bacteria decreased in obese	[60]
Hypertension	Feces	56 prehypertension 99 hypertension patients	41 healthy controls	Viruses have a superior resolution and discrimination power than bacteria for identifying hypertension More targeted virus-bacteria linkages in hypertension	[61]
Pulmonary arterial hypertension	Feces	18 type 1 PAH patients	13 reference subjects	Bacteria associated with trimethylamine/ trimethylamine *N*-oxide and purine metabolism increased in PAH. Enrichment of Enterococcal and relative depletion of Lactococcal phages in the PAH	[62]
Fatty liver	Feces	73 patients with NAFLD (29 with NAS score 0–4; 44 with NAS score 5–8)	9 individuals without liver disease and 13 patients with mild primary biliary cholangitis	Decrease in intestinal viral diversity and a significant reduction in the proportion of bacteriophages in severe NAFLD	[63]
Asthma or Pneumonia	Nasopharyngeal swabs	42 asthma children < 15 years old	78 pneumonia children < 15 years old	The Asthma group: RV-C, BoV-1 and RSV-B The pneumonia group: Bacteriophage EJ-1 and TTMV. TTV	[64]
Asthma	Nasopharyngeal swabs	24 asthma children 11 asthma children	10 healthy children 11 healthy children	Bacteriophage deficiency, while eukaryotic viral presence increased in asthma	[65]
Asthma	Nasopharyngeal swabs	15 patients with non-severe asthma 15 patients with severe asthma	12 healthy individuals	CMV and EBV more abundant in patients with asthma Bacteriophage severely reduced in patients with asthma	[66]
COPD	Nasopharyngeal swabs	63 patients from the Bergen COPD Exacerbation Study		Reduced abundance of bacteriophages in COPD patients with viral pathogens	[67]
Colorectal cancer	Feces	30 had adenomas 30 had carcinomas	30 had healthy colons	Bacteriophages including *Siphoviridae, Myoviridae* allowed the colonization of driver bacteria such as *Fusobacterium*	[68]
Colorectal cancer	Feces	11 patients in Hong Kong 46 patients in Austria 91 patients in France and Germany	112 controls in Hong Kong 63 controls in Australia 66 controls in France and Germany	Enrichment of Inovirus and Tunalikevirus in CRC development 4 taxonomic markers associated with the reduced survival rate	[69]

Abbreviation: T1D, Type 1 diabetes mellitus; T2D, Type 2 diabetes mellitus; NAFLD, Non-alcoholic fatty liver disease; PAH, Pulmonary arterial hypertension; COPD, Chronic obstructive pulmonary disease; CRC, Colorectal cancer; CMV, Cytomegalovirus; EBV, Epstein–Barr virus; RSV, respiratory syncytial virus; RV, rhinovirus.
